# Multidimensional poverty, household environment and short-term morbidity in India

**DOI:** 10.1186/s41118-017-0019-1

**Published:** 2017-06-02

**Authors:** Bidyadhar Dehury, Sanjay K. Mohanty

**Affiliations:** 1 International Institute for Population Sciences, Govandi Station Road, Deonar, Mumbai, Maharashtra 400088 India; 2 Department of Fertility Studies, International Institute for Population Sciences, Mumbai, India

**Keywords:** Multidimensional poverty, Household environmental condition, Fever, Cough, Diarrhoea, Short-term morbidity, India

## Abstract

Using the unit data from the second round of the Indian Human Development Survey (IHDS-II), 2011–2012, which covered 42,152 households, this paper examines the association between multidimensional poverty, household environmental deprivation and short-term morbidities (fever, cough and diarrhoea) in India. Poverty is measured in a multidimensional framework that includes the dimensions of education, health and income, while household environmental deprivation is defined as lack of access to improved sanitation, drinking water and cooking fuel. A composite index combining multidimensional poverty and household environmental deprivation has been computed, and households are classified as follows: multidimensional poor and living in a poor household environment, multidimensional non-poor and living in a poor household environment, multidimensional poor and living in a good household environment and multidimensional non-poor and living in a good household environment.

Results suggest that about 23% of the population belonging to multidimensional poor households and living in a poor household environment had experienced short-term morbidities in a reference period of 30 days compared to 20% of the population belonging to multidimensional non-poor households and living in a poor household environment, 19% of the population belonging to multidimensional poor households and living in a good household environment and 15% of the population belonging to multidimensional non-poor households and living in a good household environment. Controlling for socioeconomic covariates, the odds of short-term morbidity was 1.47 [CI 1.40–1.53] among the multidimensional poor and living in a poor household environment, 1.28 [CI 1.21–1.37] among the multidimensional non-poor and living in a poor household environment and 1.21 [CI 1.64–1.28] among the multidimensional poor and living in a good household environment compared to the multidimensional non-poor and living in a good household environment. Results are robust across states and hold good for each of the three morbidities: fever, cough and diarrhoea. This establishes that along with poverty, household environmental conditions have a significant bearing on short-term morbidities in India. Public investment in sanitation, drinking water and cooking fuel can reduce the morbidity and improve the health of the population.

## Introduction

Globally, three billion people are using open fires and leaky stoves, biomass (wood, animal dung and crop waste) and coal for preparing food (WHO 2011), 2.4 billion people do not have access to improved sanitation and 663 million people lack access to safe drinking water (UNICEF/WHO 2015). The Human Development Report (HDR) 2011 focused on the household’s environmental deprivations of access to basic sanitation, clean drinking water and modern cooking fuel through a poverty-focused lens (UNDP 2011). The Sustainable Development Goals (SDGs) emphasised on environmental protection at the centre of discussion and aimed at the reduction of poverty and targeted universal access to safe drinking water and basic sanitation as two of its goals (United Nations 2015). Household living condition (environment) is a proximate determinant of health that is closely associated with the economic and social well-being of the household. The global progress in household living condition, state of health and poverty reduction is uneven across and within countries.

The strategies for poverty reduction and improvement in basic household amenities^1^ have often been emphasised in national and international development agenda. For the first time, the Earth summit in 1972 highlighted the need to reduce environmental risks to achieve sustainable development and eradication of poverty (UNEP 1972). In 1987, the Brundtland Commission acknowledged the role of basic household living conditions in reducing poverty and identified poverty as the major cause and effect of global environmental problems (United Nations 1987). Goal 7 of the Millennium Declaration aimed at ensuring environmental sustainability by improving access to safe drinking water and urban sanitation by 2015. Despite these concerns, household environmental deprivations remain neglected and thus a major cause of concern in developing countries.

The household environment is directly linked to the health and productivity of the population. Evidence suggest that lack of access to improved sanitation, safe drinking water and cooking fuel remains the major cause of morbidity and mortality in developing countries (Sastry 1996; Muhuri 1996; Ayad et al. 1997; Kosek et al. 2003; Mathers et al. 2006; Black et al. 2010; Spears 2013). In 2004, an estimated 1.87 million under-five children in developing countries died due to diarrhoea (Boschi-Pinto 2008), and diarrhoea remained the second major cause of death among children (Lanata et al. 2013). Water, sanitation and hygiene account for 4% of all estimated deaths and 5.7% of the total disability-adjusted life years (Prüss et al. 2002). In developing countries, indoor air pollution causes about two million deaths annually to under-five children and is responsible for 3.7% of the loss of the total disability-adjusted life years (WHO 2007). Under-five deaths and the global disease burden can be prevented with improvement in the supply of safe drinking water, improvement in sanitation facility and maintaining hygiene (Pruss-Ustun et al. 2008).

Though India is experiencing sustained economic growth, inequality has widened in economic, social and health and health care utilisation (Stephens et al. 1997; Stephens 2011; Kjellstrom and Mercado 2008). About two thirds of the population do not have access to improved sanitation, and about one-fourth of the world’s population without improved sanitation lives in India (WHO/UNICEF 2014). Access to improved drinking water and cooking fuel is limited in India. The prevalence of waterborne diseases is high and the major cause of morbidity among children. In 2005–2006, the estimated infant mortality rate (IMR) in the lowest wealth quintile was 70.4 per thousand live births compared to 29.2 per thousand live births in the highest wealth quintile in India (International Institute for Population Sciences (IIPS) and Macro International 2007). Studies suggest that the extent of infant and under-five mortality in India is significantly greater among the multidimensional poor compared to the non-poor (Mohanty 2011). A growing number of studies have established the association of unimproved sanitation with poor health and cognitive development of children (Spears 2013; Brocklehurst 2014). Lack of access to sanitation, improved drinking water and improved cooking fuel is both economic and non-economic, two gradients of multidimensional poverty. On the economic front, an average Indian household cannot afford to build a septic toilet, which costs over a lakh of rupees, and may not afford LPG or treatment of drinking water. Poor people have limited access to basic household facilities such as improved drinking water and sanitation and clean fuel, which makes them vulnerable to health shocks. In the non-economic domain, poor awareness of hygiene and the adverse effects of unimproved drinking water and health hazards of biomass leads to reduced usage of improved sanitation, drinking water and cooking fuel.

**Fig. 1:**
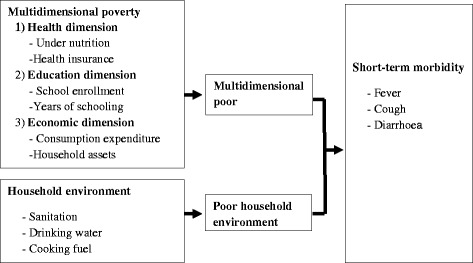
Conceptual framework of multidimensional poverty, household environment and short-term morbidity

The relationship of poverty, household environment and health is complex and context specific (Chomitz 1999; Ekbom and Bojo 1999; Bucknall et al. 2000; Bojo et al. 2001; Bosch et al. 2001; Dasgupta et al. 2005). Though attempts have been made to study on differentials in health and health care by the economic well-being of households in India, there are only a few studies that examined the linkages of multidimensional poverty, household environment and health. A schematic description of multidimensional poverty, household environment and short-term morbidity is shown in Fig. 1. The multidimensional poor are more likely to be deprived of improved sanitation, improved drinking water and improved cooking fuel, which causes adverse health situations.

The objective of this paper is to examine the linkages of multidimensional poverty, deprivation of household environment and short-term morbidities in India. This research paper has been conceptualised with the following rationales. First, the health of the population is linked to determinants of health such as improved sanitation, drinking water and cooking fuel. A poor household environment affects the health and productivity of the population adversely. Second, the multidimensional poor are likely to be living in a poor household environment, unaware of the adverse effects of a poor housing environment, and they may suffer from poor health. Hence, multidimensional poverty has a direct bearing on the health and productivity of the population. Third, existing studies on linkages of multidimensional poverty and household environment are confined to macro-level analyses and suffer from conceptualisation, data limitation and methodological deficiencies. This paper provides empirical evidence on the complex relationship between household poverty and environmental deprivation using unit data from a nationally representative population-based study in India.

## Materials and methods

### Data

The study used the unit data from the second round of the India Human Development Survey (IHDS-II), a nationally representative survey, conducted in 2011–2012. The IHDS-II interviewed 42,152 households and covered 204,569 individuals from 1503 villages and 971 urban blocks of India. A total of 14,573 households from urban and 27,579 households from rural India were covered under the survey. The IHDS-II is a population-based nationally representative survey that covered all 29 states and four union territories of India (over 99% of India’s population; however, it did not cover Andaman and Nicobar Islands and Lakshadweep). It is a multitopic survey that includes income, consumption expenditure, employment, education, fertility, reproductive health, child health, morbidities, gender relations, social capital and cognitive development of children. The survey used six sets of schedule to collect information from the community, household and individual levels. The IHDS-II collected detailed income data as well as consumption expenditure data that reflects the economic well-being of the households. We have used a set of indicators of access to improved sanitation, drinking water and cooking fuel as the household environment. The household assets and household consumption expenditure of households are used. Education of each member and the height and weight of women are also used. The survey canvassed three questions on short-term morbidities such as fever, cough and diarrhoea with a reference period of 30 days for each member of the household. A set of questions on treatment seeking and expenditure was canvassed for those who reported morbidities. However, these short-term morbidities are self-reported and have not been clinically examined. The data of the IHDS-II are of good quality and available to the public. The findings from these surveys and the unit data have been used extensively in research and policy (https://ihds.umd.edu/papers-using-ihds-public-data). The details of the survey design, sampling instrument, variables and constructed variables, and various codes used are available in the IHDS report (Desai and Vanneman 2015).

### Methodology

A number of alternative methods have been used in literature to estimate multidimensional poverty (Bourguignon and Chakravarty 2003; Alkire and Foster 2007; Calvo 2008; Wagle 2008; Jayaraj and Subramanian 2010; Mishra and Shukla 2016). Each of these methods has certain merits and limitations. Many of these methods have limitations in dealing with individual data rather than aggregate data and in decomposing poverty estimates. The Alkire-Foster (AF) method has advantages over other methods and is currently practiced in literature. We have used the AF method in estimating multidimensional poverty. The recent method of Mishra and Shukla (2016) has an advantage of addressing the inter-dependence of variables in estimating the multidimensional achievement index. We have estimated the inter-dependence of the method, and estimates are compared with the method developed by Mishra and Shukla (2016).

Table 1 provides the dimensions and variables used in estimating multidimensional poverty in India. Household poverty is measured in a multidimensional framework and includes three key dimensions of human development: education, health and economic well-being of the household. The education dimension includes two indicators—years of schooling for adult members (15 years and above) and the school enrolment status for children in the age group 6–14 years. A household is defined as poor in the education dimension if it does not have any adult member with 5 years of schooling or if any child in the school going age (6–14 years) has not been enrolled in school. The health dimension includes two indicators—undernutrition and health insurance. A household is defined as poor in the health domain if any ever-married woman aged 15–49 years in the household is undernourished (BMI <18.5) or any non-salaried household member does not have access to health insurance. Similarly, the economic dimension includes two indicators—consumption expenditure and assets. A household is defined as consumption poor if the monthly per capita consumption expenditure is below the official poverty cut-off (defined by the Planning Commission, Government of India). This is similar to the classification of households living below the poverty line. A household is defined as asset poor if the household does not own more than one of these items—television, refrigerator, telephone, bike or motorbike, and does not own a car.

**Table 1:** Mean and confidence interval of dimensional indicators of education, health and consumption expenditure in India, 2011–12

Serial no.	Dimensions	Indicators	Assigned weights	Mean (95% CI)
1	Education	School enrolment (V1): at least one child in the schoolgoing age (6–14 years) in the household currently not attending school	0.167	0.061 (0.060–0.063)
		Years of schooling (V2): no adult member (15 years and above) in the household has completed 5 years of schooling	0.167	0.138 (0.137–0.140)
2	Health	Nutrition (V3): the household has any undernourished (BMI <18.5) ever-married women (15–49 years)	0.167	0.166 (0.165–0.168)
		Health insurance (V4): the household does not have any health insurance and salaried member	0.167	0.731 (0.729–0.733)
3	Economic	Consumption expenditure (V5): if the household falls below the consumption expenditure threshold limit (official poverty line)	0.167	0.212 (0.210–0.214)
		Household assets (V6): if a household does not have more than one of television, telephone, motorbike or refrigerator, and does not own a car	0.167	0.378 (0.376–0.380)

We have estimated the multidimensional poverty index (MPI) using the dual cut-off method developed by Alkire and Foster (2007, 2011), which is disseminated in the Human Development Report (HDR) 2010 (UNDP 2010). It has used three dimensions, namely, education, standard of living and health, and assigns an equal weight to each dimension and an equal weight to each indicator within each dimension. A household gets a weighted deprivation score according to the number of weighted deprivations experienced by that household, and the total weighted deprivation score ranges between 0 and 1. A household is identified as multidimensional poor if the household’s weighted deprivation score is more than 0.33, which is one third of the total weighted deprivation score. The dimensions, indicators, weights and mean values are presented in Table 1. The weighting of variables and dimensions in the AF method is based on normative decision that assigns an equal weight to each dimension and an equal weight to variables within each dimension. The cut-off point of 0.33 is based on distribution. However, while we have used the equal weighting of dimension and indicators, a cut-off point of 0.34 is used because with that score, a household will be poor in more than one dimension.

We measured the household environmental deprivation of all the three domains—sanitation, drinking water and cooking fuel. A household is said to be poor in household environment if it does not have access to any two of the three household environmental conditions: sanitation, improved cooking fuel and improved drinking water. Access to toilet facility is considered improved sanitation, especially if the household uses the pit latrine, semi-flush (septic tank) latrine or flush toilet. Improved drinking water is defined as access to drinking water from piped tap, tube well, hand pump, covered well, rainwater and bottled water. Similarly, the household was considered not deprived of clean cooking fuel if it used improved *chulla* with chimney or fuel other than biomass (kerosene, LPG, etc.) for cooking. If the household does not use an improved source, it is referred to as unimproved. The inter-dimensional responsiveness of the three variables is shown in Appendix 1. In India, 29.7% households had access to improved drinking water, sanitation and cooking fuel, 16.2% had access to improved drinking water and sanitation only, 8.8% had access to improved drinking water and cooking fuel only and 2.4% had access to improved sanitation and cooking fuel only.

By combining multidimensional poverty and the index of household environmental deprivation, a composite index of multidimensional poverty and household environmental deprivation was computed and categorised into four categories, namely, multidimensional poor and living in a poor household environment, multidimensional non-poor and living in a poor household environment, multidimensional poor and living in a good household environment and multidimensional non-poor and living in a good household environment. Multidimensional poverty and household environmental condition are linked to three short-term morbidities—fever, cough and diarrhoea. The differentials in the prevalence of short-term morbidities are examined by multidimensional poverty and household environmental deprivation. Logistic regression is used to examine the key predictors of short-term morbidities. About 98% of the total households have information in all six indicators. We have not included households that had missing values in any of the indicators.

## Results

### Multidimensional poverty and household environmental deprivation

#### Multidimensional poverty

**Fig. 2:**
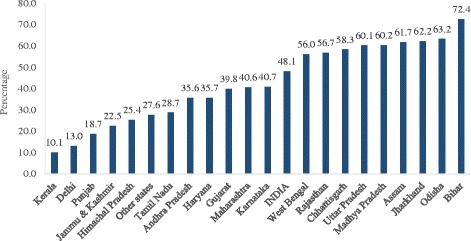
Percentage of multidimensional poor in India, 2011–2012

Figure 2 presents the estimated multidimensional poverty headcount ratio in India and its states with a population of more than four million. About 48.1% of the population was estimated as multidimensional poor in India in 2011–2012. Among the states of India, multidimensional poverty was highest in Bihar (72.4%) followed by Odisha (63.2%), Jharkhand (62.2%), Assam (61.7%), Madhya Pradesh (60.2%) and Uttar Pradesh (60.1%). Nine states (West Bengal, Assam, Madhya Pradesh, Uttar Pradesh, Rajasthan, Chhattisgarh, Odisha, Jharkhand and Bihar) had a higher percentage of multidimensional poor population than the national average. Eleven states had lower multidimensional poverty than the national average, with Kerala having the lowest (10.1%). On comparing the estimates of multidimensional poverty with poverty derived from consumption expenditure data, we found that the ranking of states differed in both the variables. The rank order correlation coefficient of consumption poverty (official estimates) and the estimated multidimensional poverty in India was 0.62.

### Validity and reliability of multidimensional poverty

**Fig. 3:**
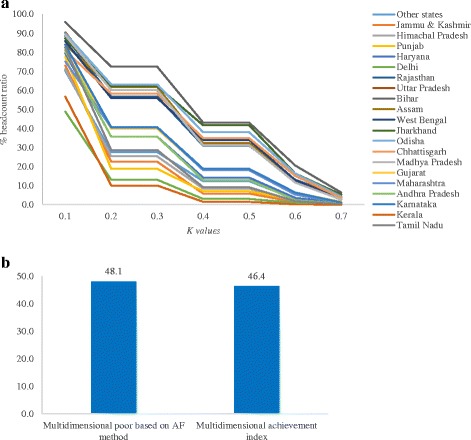
**a** Estimates of multidimensional poor with varying cut-off values (*k*), 2011–2012. **b** Multidimensional poverty estimates based on the AF method and multidimensional achievement index based on Mishra and Shukla in India, 2011–2012

We have carried out the reliability and validity of multidimensional poverty estimates. The multidimensional poverty headcount ratio varies for different values of *k* (cut-off point) for India, suggesting the reliability of the estimates. It also holds true for the states of India (Fig. 3a), suggesting that the estimates of multidimensional poverty are robust. Also, the headcount ratio estimated based on the AF method was compared with estimates from Mishra and Shukla (multidimensional achievement index). At the national level, the multidimensional poverty headcount ratio is close to the multidimensional achievement index (Fig. 3b). The correlation coefficient between the two measures is found to be −0.96 at the state level. As an external validation, the multidimensional poverty headcount ratio has also been validated with the Below Poverty Line (BPL) card and caste. Among households having a BPL card, 58.5% are multidimensional poor compared to 42.3% among households not having a BPL card, and the difference is statistically significant at *p* < 0.01. With respect to caste, about 73.7% among Scheduled Tribe were classified as multidimensional poor compared to 57% among Scheduled Caste, 48% among Other Backward Class and 34% among others.

### Household environmental deprivation index

**Fig. 4:**
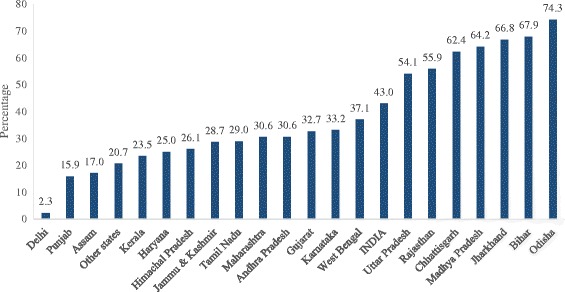
Percentage of population living in poor household environmental conditions in India, 2011–2012

Figure 4 provides the percentage of population deprived of the household environment in the states of India. We found that 43% of the population of India was living in poor household environmental conditions (does not own any two of the three or one of the three). The extent of household environment deprivation varies among the states: from 2% in Delhi to a maximum of 74% in Odisha. More than two thirds of the population of the states of Jharkhand, Bihar and Odisha were living in poor household environmental conditions. In the states of Uttar Pradesh, Rajasthan, Chhattisgarh and Madhya Pradesh, more than half of the population were living in poor household environmental conditions.

### Multidimensional poverty and household environmental deprivation index

A composite index of poverty and environmental deprivation has been computed by integrating multidimensional poverty and environmental deprivation. The composite index is categorised into four groups.

1. Multidimensional poor and living in a poor household environment
2. Multidimensional non-poor and living in a poor household environment
3. Multidimensional poor and living in a good household environment
4. Multidimensional non-poor and living in a good household environment

**Fig. 5:**
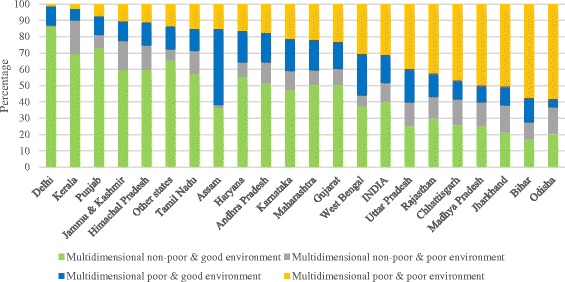
Percent distribution of multidimensional poverty and household environmental deprivation in India, 2011–2012

Results indicate that about 31% of the population of India were multidimensional poor and living in a poor household environment compared to 12% who were multidimensional non-poor and living in a poor household environment, 17% who were multidimensional poor and living in a good household environment and 40% who were multidimensional non-poor and living in a good household environment (Fig. 5). This indicates that a large proportion of multidimensional non-poor households were residing in good household environmental conditions. However, a sizeable proportion of the non-poor (12%) was also classified as poor in household environment in India. In the states of Chhattisgarh, Odisha, Jharkhand and Jammu and Kashmir, more than 15% were identified as multidimensional non-poor and also residing in poor household environmental conditions. Figures 3 and 4 show that the population belonging to non-poor households do not necessarily have access to a good household environment. The variation in state pattern is indicative that being non-poor does not necessarily mean staying in a good household environment. It depends on many factors including the availability of improved drinking water and cooking fuel and access to improved sanitation. For example, in the states of West Bengal, Odisha, Uttar Pradesh, Kerala and Bihar, hand pump and open well were predominant sources of drinking water and most of the people did not use toilet facility. Among the bigger states, seven had a higher percentage of multidimensional poor and living in a poor household environment than the national average of 31% (Fig. 5). It was highest in the state of Odisha (58%) followed by Bihar (57.5%), Jharkhand (50.4%), Madhya Pradesh (49.8%), Chhattisgarh (46.6%), Rajasthan (42.5%) and Uttar Pradesh (39.6%) and lowest in the state of Delhi (1.4%) followed by Kerala (2.7%).

### Prevalence of short-term morbidities

**Fig. 6:**
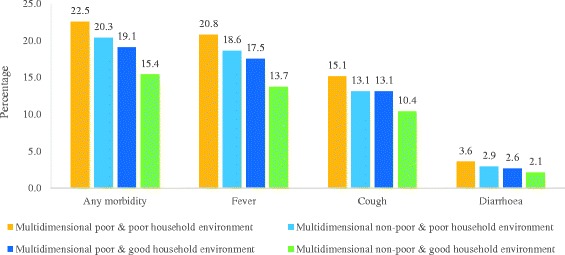
Prevalence of any short-term morbidity, fever, cough and diarrhoea, by composite index of multidimensional poverty and household environmental deprivation index in India, 2011–2012

Figure 6 presents the prevalence of any short-term morbidities, fever, cough and diarrhoea by the composite index of multidimensional poverty and household environmental deprivation, respectively. The prevalence of short-term morbidities was 22.5% among the multidimensional poor and living in a poor household environment compared to 20.3% among the multidimensional non-poor and living in a poor household environment. On the other hand, the prevalence of short-term morbidities was lowest among the multidimensional non-poor and living in a good household environment (15.4%). It may be noted that the prevalence of short-term morbidities among those who were multidimensional poor and living in a good household environment was lower than that among those who were non-poor and living in a poor household environment. This brought out the role of the household environment in determining the health of the population and indicated that the living environment is important in determining the health of the population. For example, among the multidimensional poor and living in a poor household environment, 20.8% suffered from fever compared to 18.6% of the multidimensional non-poor and living in a poor household environment, 17.5% of the multidimensional poor and living in a good household environment and 13.7% of the multidimensional non-poor and living in a good household environment. Similarly, among the multidimensional poor and living in a poor household environment, 15.1% were suffering from cough compared to 13.1% among the multidimensional non-poor and living in a poor household environment, 13.1% among the multidimensional poor and living in a good household environment and 10.4% among the multidimensional non-poor and living in a good household environment. The prevalence of diarrhoea was 3.6% among the multidimensional poor and living in a poor household environment compared to 2.9% among the multidimensional non-poor and living in a poor household environment, 2.6% among the multidimensional poor and living in a good household environment and 2.1% among the multidimensional non-poor and living in a good household environment, and these differences were statistically significant.

A similar pattern was observed by place of residence—a higher percentage of the population suffered from short-term morbidities in rural areas than in urban areas with higher variations by composite index. In rural areas, more than one fifth suffered from any short-term morbidity, 18.6% suffered from fever, 13.6% suffered from cough and 3% suffered from diarrhoea compared to 15.7, 14, 10.7 and 2.2%, respectively, in urban areas (table not shown).

The prevalence of short-term morbidities among the states is similar to the national pattern. In most of the states, the prevalence of any short-term morbidity, fever, cough and diarrhoea was higher among the multidimensional poor and living in a poor household environment. The prevalence of any short-term morbidity and diarrhoea by the composite index of multidimensional poverty and household environmental deprivation among the states of India is presented in Table 2. The table shows that the prevalence of any short-term morbidity was higher in Uttar Pradesh (30%) followed by Chhattisgarh (29.3%), Bihar (24.9%), West Bengal (21%), Punjab (20.8%) and Madhya Pradesh (20.7%). The prevalence of any short-term morbidity was lowest in Tamil Nadu (10.8%) followed by Gujarat, Karnataka, Maharashtra and Kerala (13.1%). In eight states (Uttar Pradesh, Jammu and Kashmir, Bihar, Himachal Pradesh, Andhra Pradesh, Madhya Pradesh, Kerala and Gujarat), the prevalence of any short-term morbidity was higher among the multidimensional poor and living in a poor household environment, and in six states (Punjab, Delhi, Haryana, Rajasthan, Jharkhand and Karnataka), it was higher among the multidimensional non-poor and living in a poor household environment. Similarly, in six states (Chhattisgarh, West Bengal, Odisha, Assam, Tamil Nadu and Maharashtra), it was higher among the multidimensional poor and living in a good household environment. Any short-term morbidity was lower among the multidimensional non-poor and living in a good household environment in all the major states.

**Table 2:** Prevalence of any short-term morbidity and diarrhoea by composite index of multidimensional poverty and household environmental deprivation in states of India, 2011–2012

	Any short-term morbidity					Diarrhoea				
States/India	Multidimensional poor and poor household environment	Multidimensional non-poor and poor household environment	Multidimensional poor and good household environment	Multidimensional non-poor and good household environment	All	Multidimensional poor and poor household environment	Multidimensional non-poor and poor household environment	Multidimensional poor and good household environment	Multidimensional non-poor and good household environment	All
Andhra Pradesh	23.6	21.4	20.9	16.9	19.4	1.5	2.4	1.3	2.0	1.8
Assam	6.2	10.0	16.6	15.5	14.5	1.2	6.2	2.7	3.1	2.7
Bihar	27.3	23.4	21.3	20.7	24.9	2.1	2.2	2.2	1.8	2.1
Chhattisgarh	28.7	31.0	31.7	28.4	29.3	4.0	3.6	4.3	2.5	3.6
Delhi	17.5	19.5	17.0	15.6	15.8	6.3	0.0	2.3	2.5	2.5
Gujarat	12.7	12.2	11.9	9.4	10.9	3.0	3.1	3.0	2.2	2.6
Haryana	17.7	19.0	17.8	13.3	15.4	3.1	3.4	2.9	2.4	2.7
Himachal Pradesh	23.6	17.9	20.6	18.1	19.0	4.0	2.9	2.3	4.0	3.6
Jammu and Kashmir	29.2	24.6	10.5	13.8	17.0	2.1	2.4	1.6	2.0	2.0
Jharkhand	14.1	14.6	11.6	12.5	13.5	2.5	2.8	1.4	1.6	2.2
Karnataka	11.6	12.4	12.3	11.1	11.6	1.8	2.3	1.9	1.6	1.8
Kerala	17.8	10.9	14.0	13.5	13.1	3.0	1.5	1.6	1.1	1.3
Madhya Pradesh	22.7	21.7	19.9	16.6	20.7	6.1	6.2	4.9	4.5	5.6
Maharashtra	12.9	11.8	14.2	10.9	12.0	3.3	2.7	3.3	1.5	2.3
Odisha	13.1	12.2	18.1	13.7	13.3	1.7	0.9	1.6	1.2	1.5
Punjab	25.8	30.4	21.6	19.1	20.8	1.5	1.6	1.9	1.7	1.7
Rajasthan	16.8	17.2	17.1	14.3	16.2	2.2	2.2	2.4	1.7	2.1
Tamil Nadu	13.9	12.5	14.3	8.7	10.8	2.9	2.2	1.9	1.6	1.9
Uttar Pradesh	35.1	31.4	25.0	25.4	30.0	6.7	3.8	3.5	3.5	4.8
West Bengal	19.9	19.0	23.5	20.5	21.0	1.2	1.8	1.9	1.0	1.4
Other states	28.4	28.3	15.4	12.5	16.2	6.1	5.2	3.7	3.0	3.7
Total	22.5	20.3	19.1	15.4	18.8	3.6	2.9	2.6	2.1	2.7

The prevalence of diarrhoea (Table 2) in the month preceding the survey was higher in Madhya Pradesh (5.6%) followed by Uttar Pradesh (4.8%) and Chhattisgarh (3.6%). On the other hand, the prevalence of diarrhoea was lowest in the state of Kerala (1.3%). In Uttar Pradesh, among the multidimensional poor and living in a poor household environment, the prevalence of diarrhoea was 6.7% compared to 3.8% among the multidimensional non-poor and living in a poor household environment and 3.5% each among the multidimensional poor and living in a good household environment and the multidimensional non-poor and living in a good household environment. Also, in eight states (Assam, Madhya Pradesh, Haryana, Gujarat, Jharkhand, Jammu and Kashmir, Andhra Pradesh and Karnataka), it was higher among the multidimensional non-poor and living in a poor household environment. In five states, the prevalence of diarrhoea was higher among the multidimensional poor and living in a good household environment, and in majority of the states (13 major states), the prevalence of diarrhoea was lowest among the multidimensional non-poor and living in a good household environment.

The prevalence of fever and cough by the composite index of multidimensional poverty and household environment deprivation among the states of India is presented in Table 3. The prevalence of fever was higher in Uttar Pradesh (28.2%) followed by Chhattisgarh (26.7%) and Bihar (23.3%) and lowest in Gujarat (9%). Among the multidimensional poor and living in a poor household environment, the prevalence of fever was highest in Uttar Pradesh (35%) and lowest in Assam (5.3%). In most of the states (9 states out of 20), the prevalence of fever was higher among the multidimensional poor and living in poor household environment. It was high among the multidimensional non-poor and living in a poor household environment in five states and higher among the multidimensional poor and living in a good household environment in six states.

**Table 3:** Prevalence of fever and cough by composite index of multidimensional poverty and household environmental deprivation in states of India, 2011–2012

	Fever					Cough				
States/India	Multidimensional poor and poor household environment	Multidimensional non-poor and poor household environment	Multidimensional poor and good household environment	Multidimensional non-poor and good household environment	All	Multidimensional poor and poor household environment	Multidimensional non-poor and poor household environment	Multidimensional poor and good household environment	Multidimensional non-poor and good household environment	All
Andhra Pradesh	21.5	18.4	19.7	16.1	18.0	9.4	7.9	10.3	8.7	9.0
Assam	5.7	7.2	15.9	14.7	13.8	4.4	9.3	11.7	11.6	10.5
Bihar	25.7	19.4	20.4	19.7	23.3	17.4	16.0	12.9	12.8	15.8
Chhattisgarh	26.2	29.4	29.0	24.9	26.7	20.5	24.7	23.1	20.1	21.4
Delhi	17.5	17.1	15.7	12.9	13.3	7.9	4.9	7.5	7.6	7.6
Gujarat	11.5	10.1	9.9	7.4	9.0	5.5	3.9	6.4	4.8	5.1
Haryana	15.6	16.2	16.3	10.6	13.0	7.3	12.4	8.2	6.3	7.3
Himachal Pradesh	21.1	16.1	17.7	14.2	15.7	16.1	12.9	12.7	11.2	12.2
Jammu and Kashmir	26.6	22.5	9.4	12.4	15.3	13.6	11.1	9.0	8.1	9.4
Jharkhand	12.2	13.2	10.2	11.8	12.1	8.6	8.9	8.3	7.5	8.3
Karnataka	10.4	11.3	11.3	10.0	10.5	8.6	9.4	8.6	7.9	8.3
Kerala	15.2	10.1	12.1	12.5	12.0	16.2	7.8	12.7	11.3	10.8
Madhya Pradesh	19.9	18.9	17.9	14.3	18.1	14.1	13.6	11.5	9.2	12.5
Maharashtra	12.3	11.7	13.3	10.3	11.4	8.3	8.0	7.9	5.8	6.9
Odisha	10.9	9.4	14.7	10.9	10.9	8.4	8.7	11.6	9.3	8.8
Punjab	24.9	29.9	20.6	17.2	19.2	19.3	26.0	15.7	14.0	15.6
Rajasthan	15.2	16.1	15.7	12.9	14.7	11.6	12.6	11.9	10.6	11.5
Tamil Nadu	12.3	11.0	13.5	7.3	9.4	10.6	9.0	9.9	6.5	7.9
Uttar Pradesh	33.5	30.5	23.0	22.9	28.2	24.9	20.0	18.7	19.8	21.6
West Bengal	17.8	16.4	20.7	17.5	18.3	17.7	15.7	20.8	18.0	18.5
Other states	26.8	27.5	12.5	11.1	14.6	21.3	14.5	9.2	8.4	10.7
Total	20.8	18.6	17.5	13.7	17.1	15.1	13.1	13.1	10.4	12.6

Among the major states of India, the prevalence of cough (Table 3) was highest (21.6%) in Uttar Pradesh followed by Chhattisgarh (21.4%), West Bengal (18.4%) and Bihar (15.8%). It was lowest in Gujarat (5.1%) followed by Maharashtra (6.9%), Haryana (7.3%) and Delhi (7.6%). Among the multidimensional poor and living in a poor household environment, the prevalence of cough was higher in Uttar Pradesh (24.9%) followed by Chhattisgarh, Punjab, West Bengal and Bihar. It was lowest in Assam (4.4%) followed by Gujarat, Haryana and Delhi. The prevalence of cough was high among the multidimensional poor and living in a poor household environment in nine states, high among the multidimensional non-poor and living in a poor household environment in six states and high among the multidimensional poor and living in a good household environment in five states.

### Association of short-term morbidities by poverty and household environment

**Fig. 7:**
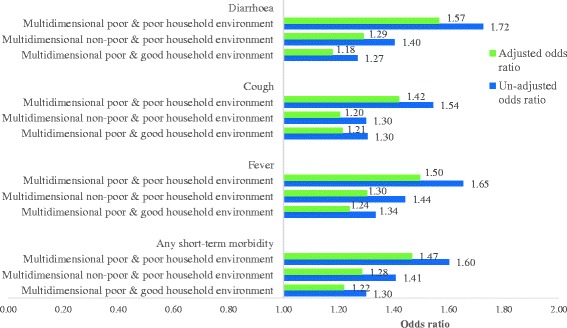
Un-adjusted and adjusted odds ratios of short-term morbidities (any short-term morbidity, fever, cough and diarrhoea) in India, 2011–2012. Note: dependent variables are fever (0 = no, 1 = yes); cough (0 = no, 1 = yes) and diarrhoea (0 = no, 1 = yes). Odds are adjusted for age, sex, caste, religion and place of residence. Odds ratios are statistically significant (*p* < 0.01)

Logistic regression has been used to examine the association of multidimensional poverty, household environment and short-term morbidities. The dependent variables are as follows: any of the three short-term morbidities (cough, fever and diarrhoea) and each of the morbidities (categorised as dichotomous: 0 = no and 1 = yes). The independent variables are demographic and socio-economic covariates: age (continuous), sex, caste (Other Backward Class (OBC), Scheduled Caste (SC), Scheduled Tribe (ST) and others), religion (Hindu, Muslim and others) and place of residence (rural and urban). The odds of short-term morbidity by categories of the composite index of multidimensional poverty and household environmental condition in India are presented in Fig. 7. Adjusted and unadjusted odds ratios are presented for each of the three morbidities by controlling for other demographic and socio-economic characteristics. Those who were multidimensional non-poor and living in a good household environment are taken as the reference category. Both adjusted and unadjusted odds ratios show a similar pattern, but the adjusted odds ratio is lower than the unadjusted odds ratio. Compared to the reference category, the odds of any short-term morbidity was 1.47 [95% CI 1.40–1.53] among the multidimensional poor and living in a poor household environment, 1.28 [CI 1.21–1.37] among the multidimensional non-poor and living in a poor household environment and 1.22 [CI 1.16–1.28] among the multidimensional poor and living in a good household environment. The coefficients are similar for fever, cough and diarrhoea. Compared to the multidimensional non-poor and living in a good household environment, the odds of suffering from fever was 1.49 [95% CI 1.42–1.57] among the multidimensional poor and living in a poor household environment, 1.30 [CI 1.22–1.39] among the multidimensional non-poor and living in a poor household environment and 1.24 [CI 1.18–1.30] among the multidimensional poor and living in a good household environment. The odds of suffering from cough was 1.42 [CI 1.34–1.50] among the multidimensional poor and living in a poor household environment, 1.20 [CI 1.12–1.29] among the non-poor and living in a poor household environment and 1.21 [CI 1.15–1.28] among the multidimensional poor and living in a good household environment. The prevalence of diarrhoea was 56% more among the multidimensional poor and living in a poor household environment, 29% more among the multidimensional non-poor and living in a poor household environment and 18% more among the multidimensional poor and living in a good household environment compared to the multidimensional non-poor and living in a good household environment. The odds of short-term morbidities are significant for all categories of the composite index. The unadjusted odds ratio and adjusted odds ratio for short-term morbidities (any short-term morbidity, fever, cough and diarrhoea) by the composite index of multidimensional poverty and household environmental condition in the big states of India are presented in Tables 4 and 5. In all the states, except Assam, the odds of having any short-term morbidity was higher among the multidimensional poor and living in a poor household environment compared to the multidimensional non-poor and living in a good household environment. In most of the states, the odds were found significant. This validates that household environmental living conditions are a critical determinant of morbidity and health after controlling for other covariates.

**Table 4:** Un-adjusted and adjusted odds ratios of any short-term morbidity in the states of India, 2011–2012

	Any short-term morbidity						Diarrhoea						
	Un-adjusted odds ratio			Adjusted odds ratio			Un-adjusted odds ratio			Adjusted odds ratio			
States/India	Multidimensional poor and poor household environment	Multidimensional non-poor and poor household environment	Multidimensional poor and good household environment	Multidimensional poor and poor household environment	Multidimensional non-poor and poor household environment	Multidimensional poor and good household environment	Multidimensional poor and poor household environment	Multidimensional non-poor and poor household environment	Multidimensional poor and good household environment	Multidimensional poor and poor household environment	Multidimensional non-poor and poor household environment	Multidimensional poor and good household environment	Number
Andhra Pradesh	1.52***	1.34***	1.30***	1.48***	1.38***	1.26**	0.74	1.22	0.64*	0.91	1.48	0.68	8686
Assam	0.36***	0.61	1.09	0.35***	0.56	1.04	0.38*	2.08	0.87	0.31**	1.32	0.77	4352
Bihar	1.44***	1.18	1.04	1.30***	1.11	0.97	1.19	1.22	1.24	0.88	1.01	1.03	8241
Chhattisgarh	1.02	1.14	1.17	1.10	1.11	1.19	1.61**	1.43	1.74**	1.48	1.34	1.65*	6293
Delhi	1.15	1.31	1.11	1.01	1.29	1.01	2.63*	–	0.91	2.36	–	0.78	4415
Gujarat	1.40***	1.34**	1.30**	1.12	1.07	1.18	1.38*	1.43	1.38	1.25	1.22	1.32	9115
Haryana	1.41***	1.53***	1.41***	1.15	1.32**	1.27**	1.34	1.46	1.23	1.49*	1.62**	1.30	9275
Himachal Pradesh	1.40***	0.99	1.18	1.35**	0.96	1.16	1.00	0.71	0.57**	1.08	0.70	0.62**	6586
Jammu and Kashmir	2.57***	2.04***	0.73*	2.00***	1.41**	1.01	1.09	1.26	0.83	0.90	0.91	1.11	3788
Jharkhand	1.14	1.19	0.91	1.13	1.04	0.99	1.57	1.80	0.86	2.32**	1.85	1.11	4358
Karnataka	1.05	1.14	1.13	0.98	1.11	1.07	1.15	1.50	1.21	0.91	1.24	1.05	17,430
Kerala	1.38	0.78**	1.04	1.59*	0.87	1.07	2.69*	1.37	1.43	2.78*	1.35	1.46	6439
Madhya Pradesh	1.48***	1.40***	1.25***	1.32***	1.23**	1.18*	1.36***	1.39**	1.10	1.21	1.21	1.02	15,062
Maharashtra	1.20**	1.09	1.34***	1.12	1.08	1.26**	2.30***	1.85***	2.26***	1.44	1.28	1.70**	15,498
Odisha	0.95	0.87	1.39*	1.26**	1.08	1.55**	1.42	0.73	1.36	1.60	0.78	1.43	9782
Punjab	1.47***	1.85***	1.17	1.26	1.57***	1.05	0.91	0.97	1.12	0.79	0.94	0.94	8393
Rajasthan	1.21***	1.25***	1.23**	1.12	1.19*	1.16*	1.32*	1.29	1.40*	1.33	1.33	1.26	14,106
Tamil Nadu	1.71***	1.51***	1.76***	1.60***	1.40**	1.75***	1.92**	1.40	1.24	2.19***	1.60	1.24	7413
Uttar Pradesh	1.59***	1.35***	0.98	1.69***	1.45***	1.00	1.98***	1.09	1.00	1.96***	1.10	0.95	21,253
West Bengal	0.96	0.91	1.19**	0.83**	0.81	1.07	1.16	1.73	1.80**	1.19	1.74	1.73**	10,392
Other states	2.77***	2.76***	1.27**	2.03***	2.02***	1.28*	2.10**	1.77*	1.24	1.89*	1.49	1.23	8231

***p < 0.01, **p < 0.05, *p < 0.1

**Table 5:** Un-adjusted and adjusted odds ratios of fever and cough in the states of India, 2011–2012

	Fever						Cough						
	Un-adjusted odds ratio			Adjusted odds ratio			Un-adjusted odds ratio			Adjusted odds ratio			
States/India	Multidimensional poor and poor household environment	Multidimensional non-poor and poor household environment	Multidimensional poor and good household environment	Multidimensional poor and poor household environment	Multidimensional non-poor and poor household environment	Multidimensional poor and good household environment	Multidimensional poor and poor household environment	Multidimensional non-poor and poor household environment	Multidimensional poor and good household environment	Multidimensional poor and poor household environment	Multidimensional non-poor and poor household environment	Multidimensional poor and good household environment	Number
Andhra Pradesh	1.43***	1.17	1.28***	1.37***	1.20	1.24**	1.09	0.47	0.90	1.13	0.97	1.21	8686
Assam	0.35***	0.45	1.10	0.33***	0.40	1.03	0.35	0.00	0.78	0.33***	0.65	0.98	4352
Bihar	1.42***	0.99	1.05	1.30**	0.94	0.99	1.43	0.00	1.30	1.31**	1.24	0.95	8241
Chhattisgarh	1.07	1.26**	1.23*	1.10	1.19	1.21	1.03**	0.74	1.31	1.22*	1.37**	1.29**	6293
Delhi	1.43	1.39	1.26*	1.26	1.41	1.15	1.05	0.92	0.63	0.90	0.59	0.87	4415
Gujarat	1.63***	1.42**	1.38***	1.22	1.08	1.21	1.16	0.33	0.81**	0.89	0.66	1.24	9115
Haryana	1.55***	1.62***	1.63***	1.19	1.34**	1.42***	1.19***	0.15	2.12**	1.05	1.94***	1.24*	9275
Himachal Pradesh	1.62***	1.16	1.30**	1.55***	1.12	1.27**	1.52	0.00	1.18	1.43***	1.14	1.12	6586
Jammu and Kashmir	2.57***	2.05***	0.73*	1.99***	1.45**	0.97	1.78**	0.01	1.41	1.58**	1.19	1.26	3788
Jharkhand	1.04	1.14	0.85	0.98	0.99	0.88	1.16	0.36	1.21	1.06	1.03	1.12	4358
Karnataka	1.05	1.15	1.14*	0.97	1.12	1.08	1.10	0.33	1.21	1.03	1.17	1.05	17,430
Kerala	1.25	0.78**	0.96	1.41	0.87	0.99	1.52***	0.11	0.67	1.84**	0.78**	1.20	6439
Madhya Pradesh	1.49***	1.40***	1.31***	1.35***	1.25**	1.25**	1.61***	0.00	1.54**	1.40***	1.38***	1.19	15,062
Maharashtra	1.23**	1.16	1.34***	1.17	1.16	1.27**	1.48***	0.00	1.43***	1.28*	1.32*	1.25*	15,498
Odisha	1.00	0.85	1.41	1.24*	1.00	1.53*	0.90	0.34	0.93	1.30*	1.24	1.46**	9782
Punjab	1.60***	2.06***	1.25**	1.30	1.67***	1.10	1.47***	0.01	2.16	1.28	1.86**	1.03	8393
Rajasthan	1.21***	1.30***	1.26***	1.14*	1.25**	1.20**	1.11**	0.13	1.22	1.11	1.24	1.11	14,106
Tamil Nadu	1.78***	1.57***	1.98***	1.70***	1.50*	1.95***	1.70**	0.00	1.42***	1.56***	1.27	1.56***	7413
Uttar Pradesh	1.70***	1.48***	1.01	1.81***	1.60***	1.03	1.34	0.00	1.01	1.46***	1.12	0.95	21,253
West Bengal	1.02	0.93	1.23***	0.85	0.80*	1.07	0.99	0.85	0.85**	0.85*	0.75**	1.07	10,392
Other states	2.93***	3.04***	1.14	2.00***	2.10***	1.08	2.96**	0.00	1.85	2.10	1.35***	1.04	8231

***p < 0.01, **p < 0.05, *p < 0.1

## Discussion and conclusion

The aim of this paper is to understand the linkages between multidimensional poverty, household environmental condition and short-term morbidity in India using data from a nationally representative population-based survey. Multidimensional poverty is measured using the Alkire-Foster method, and household environment is measured using access to improved sanitation, drinking water and cooking fuel. Short-term morbidity is limited to fever, cough and diarrhoea. While we used the Alkire-Foster method and the same dimensions of the global MPI, the selection of indicators was context specific and depended on availability in the data set.

Our results suggest that about half of India’s population is multidimensional poor, and the estimate of multidimensional poor varies across states. States with a higher proportion of multidimensional poor also have lower access to improved drinking water, sanitation and cooking fuel. Focusing on states with a high prevalence of multidimensional poverty could help reduce household environmental deprivation of improved water, sanitation and cooking fuel. Poor sanitation is also associated with a vicious circle of disease and linked to poverty and ignorance. It may be mentioned that improving sanitation has been accorded high priority in policy initiatives. The Total Sanitation Campaign (TSC) conducted in rural areas from 1999 to 2012 was not very successful. Evaluation of the TSC suggests that the success of the program in selected districts was due to a comprehensive approach of demand creation for sanitation, development of technological solutions tailored to consumer preference and focusing on changing behaviour (World Bank, Water and Sanitation Program (WSP) 2013). The *Swasth Bharat Abhiyan* (Clean India, Healthy India), initiated in 2014, aimed at eliminating open defecation, eradicating manual scavenging and generating awareness among citizens about sanitation and its linkages with public health. Intervention studies also showed modest reduction in open defecation owing to the TSC (Patil et al. 2014). Studies also suggest that a significant proportion of rural population revealed preference for open defecation even with access to toilets (Gupta et al. 2014). Creating public awareness to use toilet facility and increased monetary incentive to build toilets are suggested. This could be achieved by strong political will involving central, state and local governments and by involving the electronic and the print media.

Similarly, water scarcity is a challenging issue in many parts of the country. It involves a wide range of issues such as supply and demand of water, the quality of drinking water and management of drinking water. In many parts of India, water supply is scarce, depends on the regularity and quantity of rainfall and is regulated by the local/state government. Besides, the drinking water supply is not always safe and often suffers from contamination. Waterborne diseases, leading to fever and diarrhoea, are common causes of morbidity in many parts of India. Public provision of tap water, efficient management of water and monitoring the quality of water would be helpful. With respect to cooking fuel, a large proportion of households of India use unimproved cooking fuel (biomass), which are hazardous to the health of the population. The recent drive to increase the coverage of LPG across the country is a welcome step. The coverage of LPG has increased from 17.5% in 2001 to 28.5% in 2011.

Our results confirmed a higher prevalence of short-term morbidities among those who were multidimensional poor and living in a poor household environment compared to the other households. This shows that along with poverty, household environmental conditions have an important effect on the health of the population in general and short-term morbidities in particular. Providing access to improved sanitation, drinking water and cooking fuel requires a multipronged strategy that will certainly improve the health of the population.

## Appendix 1

**Table 6:** Inter-dimensional responsiveness (%) in household environment (sanitation, drinking water and cooking fuel) in states of India, 2011–2012

States/India	All three	Sanitation	Drinking water	Fuel	Sanitation and drinking water	Drinking water and fuel	Sanitation and fuel	None of these	Number
Andhra Pradesh	39.7	1.4	28.6	1.2	11.8	11.2	3.2	2.9	2162
Assam	44.2	1.6	15	0.3	33.3	3.8	1.6	0.3	980
Bihar	10.7	0.9	67.2	0.0	17.2	2.6	0.1	1.3	1529
Chhattisgarh	14.0	2.5	49.6	0.1	19.7	1.8	1.5	10.8	1321
Delhi	76.4	0.2	1.5	0.2	3.1	11.5	7.0	0.1	896
Gujarat	41.2	1.1	25.6	1.7	16.2	8.9	1.4	3.8	1888
Haryana	33.7	1.4	22.3	0.1	38.3	2.0	0.3	2.0	1774
Himachal Pradesh	25.5	5.1	17.6	0.3	44.7	3.3	1.2	2.5	1476
Jammu and Kashmir	42.8	3	17.3	1.1	15.3	6.1	2.5	12.1	720
Jharkhand	10.2	2.1	49.7	2.7	10.4	9.1	1.8	14.1	853
Karnataka	37.3	1.4	25.6	1.5	7.6	18.2	4.0	4.5	3810
Kerala	38.4	20.3	0.4	0.2	15.3	0.6	24.5	0.4	1544
Madhya Pradesh	18.0	2.6	45.3	0.7	11.2	4.3	0.7	17.2	3122
Maharashtra	41.4	1.2	24.1	0.6	14.5	11.6	0.7	6.0	3287
Odisha	11.2	3.9	56.8	0.2	10.4	1.2	1.7	14.9	2057
Punjab	49.5	1.2	15.1	0.0	29.1	3.3	1.4	0.5	1700
Rajasthan	20.2	2.6	39.7	2.2	11.7	9.6	1.0	13.1	2697
Tamil Nadu	39.8	0.9	24.8	1.7	5.2	20.9	3.5	3.3	1967
Uttar Pradesh	17.0	0.6	50.5	0.6	14.7	12.3	1.0	3.2	3817
West Bengal	25.4	2.6	30.4	0.1	34.9	2.4	0.6	3.6	2430
Other states	49.2	5.8	11.2	0.2	21.0	5.8	3.9	2.8	1848
Total	29.7	2.3	34.2	0.8	16.2	8.8	2.4	5.5	41,878

## Footnotes

1 Basic household amenities include basic sanitation, clean cooking fuel and safe drinking water
